# Association between lifestyle, parental smoke, socioeconomic status, and academic performance in Japanese elementary school children: the Super Diet Education Project

**DOI:** 10.1186/s12199-019-0776-x

**Published:** 2019-04-09

**Authors:** Masaaki Yamada, Michikazu Sekine, Takashi Tatsuse, Yukiko Asaka

**Affiliations:** Department of Epidemiology and Health Policy, Graduate School of Medicine and Pharmaceutical Sciences, University of Toyama, 2630 Sugitani, Toyama, 930-0194 Japan

**Keywords:** Academic performance, Secondhand smoke, Socioeconomic status, Super Diet Education Project, Wakeup time

## Abstract

**Background:**

Health and education are closely linked. However, few studies have explored the correlates of children’s academic performance in Japan. We aimed to investigate comprehensively the associations of low academic performance among school children with lifestyles, parental smoke, and socioeconomic status.

**Methods:**

In 2016, children aged 6 to 13 years from the Super Diet Education School Project were surveyed using questionnaires. The survey explored the lifestyles and subjective academic performance of 1663 children and asked their parents about parental smoke and subjective socioeconomic status. Academic performance and socioeconomic status were divided into three levels. Then, we defined subjective academic performance in the lower two levels as low academic performance. The odds ratios (OR) were analyzed by logistic regression analysis.

**Results:**

Among all participants, 299 (18.0%) children reported low academic performance. In general, low academic performance was significantly associated with late wakeup time (OR = 1.36 for 6:30 to < 7 a.m. and OR = 2.48 for ≥ 7 a.m.), screen time ≥ 2 h (OR = 1.35), studying at home < 1 h (OR = 1.82), paternal smoke (OR = 1.47), maternal smoke (OR = 1.87), and low socioeconomic status (OR = 1.48). Analyses stratified by grade showed stronger associations between academic performance and socioeconomic status in senior (OR = 1.62 for middle, OR = 1.52 for low in grades 4 to 6) than in junior children (OR = 1.15 for middle, OR = 1.38 for low in grades 1 to 3).

**Conclusions:**

Children’s lifestyles, parental smoke, and socioeconomic status were significantly associated with low academic performance among Japanese children. Parents and health care providers should take these findings into consideration to prevent children from having low academic performance.

## Introduction

Health and education are closely linked. It is established that academic success in childhood is a strong predictor of future wealth, productivity, and health [1, 2], while children in families with low educational opportunities or socioeconomic status (SES) are at high risk for negative health and developmental outcomes [3, 4]. The health inequalities due to SES have emerged as a public health concern with regard to social instability and national health expenditure [5]. Given these facts, educational authorities and public health decision makers must take children’s academic performance (AP) into consideration to prevent chronic disease and reduce health inequalities.

According to previous studies, children’s AP is associated with sex; obesity; lifestyles such as physical activity, screen time, and diet; and SES [6–9]. Especially, in Western countries, SES is a well-recognized factor affecting children’s AP [10–12]. However, few studies on SES and AP have been conducted in Japan. This can be explained by the fact that historically, there has been strong concern about ranking schools by academic performance in the society and academic institutions, because identifying schools ranked low with AP would lead to prejudice of class or regional discrimination [12]. It was not until the early 2000s, when job instability and economic inequality came to the fore, that a few findings about SES and AP emerged in Japan [12]. Nevertheless, Japan still lacks studies on AP and SES.

The Japan Education Longitudinal Study demonstrated that the AP of 6th-grade elementary school children was positively associated with household income [13]. The Nippon Foundation, which conducted a population study with about 25,000 children in Osaka, also reported that children in households on welfare had lower academic scores than others and that wider score differences were seen in children aged 10 or older [14]. However, these studies mainly focused on study-related factors such as income, parental academic achievement, cram school, and study habit. Other lifestyles of children and parents that can affect children’s cognitive development were not included.

In children, secondhand smoke is thought to increase the risk of physical health problems, such as respiratory disorders and sudden death syndrome, and is also known to be associated with psychosocial problems, such as mental and neuro-behavioral developmental disorders [15, 16]. In addition, a review article showed a significant association between parental smoke and cognitive functioning among children [17]. Recent years have seen legal advances to combat secondhand smoke exposure in Japan [18]; however, no research, to our knowledge, about children’s AP and secondhand smoke to support the governmental legislation has been conducted in the country.

Thus, the purposes of our study were as follows: (1) to comprehensively investigate the associations of children’s AP with their overall lifestyles, parental smoke, and SES in Japan and (2) to conduct grade-stratified analyses to identify whether the strength of the association with AP varies according to age.

## Methods

### Participants and the Super Diet Education (*Shokuiku*) Project

The Super Diet Education (*Shokuiku*) Project was a food education project supported by the Japanese Ministry of Education, Culture, Science and Technology [19, 20]. The overall purposes of the project were to promote healthy lifestyles in school children and improve their health through food education. The project was evaluated using repeated questionnaire surveys before and after food education. The baseline survey (phase 1) was conducted before food education in May 2014. The follow-up surveys were conducted after food education in December 2014 (phase 2) and January 2016 (phase 3). We obtained detailed information on children’s lifestyles, AP, parental smoke, and subjective SES from phase 3 and conducted a cross-sectional study. Participants were 2129 children aged 6 to 13 years who belonged to five public elementary schools in Takaoka city, Toyama, Japan. Takaoka is the second largest city in Toyama Prefecture, which is located approximately in the center of Honshu, Hokuriku district. The city’s population is approximately 170,000.

Twenty children were excluded owing to parents’ illiteracy in Japanese. A total of 1986 children agreed to participate in our survey and returned the questionnaires (response rate 94.2%).

### Instruments

We distributed a set of questionnaires to all children via their schools. Teachers explained the purpose of the survey, and the children and their parents completed the questionnaires and returned them to their schools. The questionnaires for children included information on sex; grade (from 1 to 6); overall lifestyles such as diet, sleep, physical activity, screen time, and study duration at home; cram school; and subjective AP. Questionnaires for parents included assessments of parental smoke, subjective SES, and their children’s anthropometry data.

Children’s overall lifestyles (breakfast, physical activity, screen time, and sleep and study habits), daytime sleepiness, and AP were assessed. Responses to breakfast were dichotomized into two groups as follows: “having breakfast every day” or “sometimes to usually skipping breakfast.” Physical activity was divided into three levels: “very often,” “often,” or “rarely to almost never.” We also asked for information on the total screen time and study duration at home on a weekday. Screen time included TV and DVD viewing, gaming, and Internet use. Answers on screen time were categorized from 1 to 6, as follows: 1, no or almost no screen time; 2, < 1 h; 3, 1 to < 2 h; 4, 2 to < 3 h; 5, 3 to < 4 h; and 6, ≥ 4 h. Referring to a report from the Japan Pediatric Association, which recommends a restriction of total screen time to within 2 h a day [21], children were then divided into two groups:“< 2 h” or “≥ 2 h.” The study duration at home was divided into “almost none,” “< 1 h,” “1 to < 2 h,” “2 to < 3 h,” and “≥ 3 h.” Then, the duration was dichotomized into “< 1 h” or “≥ 1 h.” Response to wakeup time and bedtime were categorized into three groups: “< 6:30 a.m.”, “6:30 to < 7:00 a.m.”, and “≥ 7:00 a.m.” for wakeup time, and “< 10:00 p.m.”, “10:00 to < 10:30 p.m.”, and “≥ 10:30 p.m.” for bedtime. In accordance with reports that the average bedtimes for Japanese elementary school children were 9:44 p.m. for grade 3 and 10:04 p.m. for grade 6 [22], we defined “≥ 10:00 p.m.” as late bedtime for children in grades 1 to 3, while “≥ 10:30 p.m.” was defined as late for grades 4 to 6. The validity of the lifestyle questionnaire that assessed physical activity and sleep was examined in our previous study [23]. In that study, frequent physical activity was significantly correlated with increased energy expenditure, mean steps, and mean activity count per day on the Actiwatch (*p* < 0.05 for a linear trend test). The correlation coefficient between subjective and objective records was 0.97 (*p* < 0.001) for assumed amount of sleep [24].

From the parents, we obtained children’s height and weight, which were measured in January 2016 by trained school nurses. The percentage of obesity is usually assessed in the School Health Statistics Research in Japan [25]. Age-, sex-, and height-based ideal body weight (BW; kg) was calculated based on the national statistics [25], from which the percentage of deviance from the ideal BW was calculated [(individual BW − ideal BW)/ideal BW]. The percentage of obesity was reported to have a strong correlation with body mass index (BMI) Z score (*R*^2^ = 0.957 for boys and 0.949 for girls) [26]. Children who are more than 20% over their ideal BW are regarded as obese in Japan, which Isojima et al. reported to be almost equivalent to those above the 95th percentile of their age- and sex-specific BMI [26].

Regarding subjective AP, we asked children, “Are you able to understand school lesson well?” The responses were on a 5-point scale and collapsed into three: “well or relatively well,” “relatively poor or poor,” and “neither.” Then, the latter two responses were defined as low AP. Self-reported grades and scores have previously been reported to be generally accurate [27]. Subjective AP is widely used as a feasible surrogate variable [1, 8, 28], especially in Japan, where access to children’s actual academic data for research is limited [12].

The question on parental smoking was answered with “yes” or “no.” Regarding SES, we asked parents about their subjective family affluence. The responses to the question were divided into three categories: “affluent,” “no affluent,” and “neither,” and we defined them as “high,” “low,” and “middle” SES, respectively.

### Data analysis

Taking children’s grade (age) differences for lifestyles into consideration, we divided them into two groups, junior (1–3) and senior (4–6) grades, for descriptive data. A chi-squared test was conducted to compare variables by group. Next, univariate and multivariate logistic regression analyses were conducted to evaluate the associations between children’s lifestyles, obesity, parental smoke, SES, and low AP. Following the overall analysis, analyses stratified by grade (junior and senior) were performed to identify the age-related differences in the association with AP. The odds ratios (OR) and 95% confidence intervals (CI) were calculated. Finally, as a post hoc test, we calculated the rates of lifestyles by SES. Analyses were conducted using the Statistical Package for Social Scientists (SPSS) version 25.0 J (SPSS, Chicago, IL, USA). A two-tailed *p* value of less than 0.05 was considered statistically significant.

## Results

Out of the 1986 participants who returned their questionnaires, 1663 school children [78.1%; 829 boys and 834 girls, mean 9.5 years old (1.75 SD)] who answered all the questions relevant to the present study were included in our analyses. In total, 18.0% of children (*n* = 299), who answered “relatively poor or poor AP” or “neither,” were defined as displaying low AP. In comparison with children in junior grades (1–3), those in senior grades were more likely to skip breakfast, have longer screen time, daytime sleepiness, later bedtime, and long study duration at home and were less likely to be physically active. There were no significant differences in wakeup time, obesity, cram school, parental smoke, and SES (Table 1). Compared to the children included in our analyses, those who were excluded (*n* = 466, 21.9%) were more likely to have low AP (30.4% vs 18.0%), low SES (44.0% vs 24.5%), screen time ≥ 2 h (50.0% vs 38.9%), and maternal smoke (23.6% vs 10.9%).

**Table 1:** Characteristics of participants by grade

	Junior (1–3)	Senior (4–6)	
Total *n* = 1663	*n* = 826 (%)	*n* = 837 (%)	*P* value
Sex (boy)	50.1	49.6	0.83
Wakeup time on week day			
< 6:30 a.m.	52.8	48.6	0.07
6:30 to < 7:00	44.2	46.6	
≥ 7:00	3.0	4.8	
Skipping breakfast (yes)	4.5	8.7	< 0.001
Screen time on week day (≥ 2 h)	34.5	43.2	< 0.001
Physical activity			
Very often	28.3	28.7	< 0.001
Often	48.8	39.7	
Rarely	22.9	31.7	
Bedtime on week day			
< 10 p.m.	88.6	53.7	< 0.001
10 to < 10:30	9.2	31.2	
10:30 or later	2.2	15.2	
Daytime sleepiness (yes)	12.8	20.9	< 0.001
Obesity (yes)	8.2	7.8	0.73
Cram school (yes)	23.0	27.0	0.06
Study duration at home (< 1 h)	74.8	50.3	< 0.001
Paternal smoke (yes)	42.1	46.2	0.09
Maternal smoke (yes)	10.2	11.7	0.32
Subjective SES			
High	28.3	26.9	0.73
Middle	47.8	47.9	
Low	23.8	25.2	
Subjective academic performance			0.20
Well or relatively well	82.4	81.6	
Neither	11.9	14.1	
Relatively poor or poor	5.7	4.3	

Pearson chi-square test

*SES,* socioeconomic status

In Table 2, logistic regression analyses were performed to determine the association between children’s lifestyles, parental smoke, SES, and low AP in overall children. For univariate analysis, late wakeup time (OR 1.49; 95% CI, 1.15–1.93 for 6:30 to < 7:00 and OR 2.77; 95% CI, 1.59–4.81 for ≥ 7:00), skipping breakfast (OR 1.70; 95% CI, 1.09–2.65), screen time ≥ 2 h (OR 1.59; 95% CI, 1.24–2.05), studying at home < 1 h (OR 1.89; 95% CI, 1.43–2.50), paternal smoke (OR 1.66; 95% CI, 1.29–2.14), maternal smoke (OR 2.34; 95% CI, 1.66–3.29), and SES (OR 1.43; 95% CI, 1.04–1.97 for middle SES and OR 1.62; 95% CI, 1.14–2.32 for low SES) were associated with low AP in children. In the multivariate analysis, late wakeup time (OR 1.36; 95% CI, 1.03–1.48 for 6:30 to < 7:00 and OR 2.48; 95% CI, 1.36–4.05 for ≥ 7:00), screen time ≥ 2 h (OR 1.35; 95% CI, 1.04–1.76), studying at home < 1 h (OR 1.82; 95% CI, 1.34–2.49), paternal smoke (OR 1.47; 95% CI, 1.12–1.92), maternal smoke (OR 1.87; 95% CI, 1.29–2.72), and low SES (OR 1.48; 95% CI, 1.03–2.14) had significant associations with low AP. Less physically active children were likely to have low AP; however, the association was not significant.

**Table 2:** Overall analyses on low academic performance *n* = 1663

	Low academic	Univariate		Multivariate^a^	
	%	OR (95% CI)	*p*	OR (95% CI)	*p*
Sex (girl/boy)	17.4/18.6	0.92 (0.72–1.19)	0.53	1.00 (0.77–1.31)	0.98
Senior grade (4–6)/junior (1–3)	18.4 / 17.6	1.06 (0.82–1.36)	0.65	1.11 (0.84–1.45)	0.47
Wakeup time on week day					
< 6:30 a.m.	14.7	1		1	
6:30 to < 7:00	20.4	*1.49 (1.15–1.93)*	*< 0.01*	*1.36 (1.03–1.78)*	*0.03*
**≥** 7:00	32.3	*2.77 (1.59–4.81)*	*< 0.001*	*2.48 (1.36–4.50)*	*< 0.01*
Skipping breakfast (yes/no)	26.4/17.4	*1.70 (1.09–2.65)*	*0.02*	1.10 (0.68–1.78)	0.71
Screen time on week day (≥ 2 h/< 2 h)	22.3/15.3	*1.59 (1.24–2.05)*	*< 0.001*	*1.35 (1.04–1.76)*	*0.03*
Physical activity					
Very often	15.6	1		1	
Often	18.4	1.22 (0.89–1.66)	0.22	1.30 (0.94–1.79)	0.11
Rarely	19.8	1.34 (0.95–1.88)	0.09	1.33 (0.93–1.90)	0.11
Bedtime on week day (late/not late) late: **≥** 10 for junior, and **≥** 10:30 for senior grades children	20.8/17.5	1.23 (0.87–1.76)	0.24	0.87 (0.59–1.28)	0.49
Daytime sleepiness (yes/no)	21.0/17.4	1.27 (0.92–1.74)	0.15	1.10 (0.78–1.54)	0.58
Obesity (yes/no)	20.3/17.8	1.18 (0.76–1.83)	0.47	1.07 (0.67–1.70)	0.78
Cram school (no/yes)	19.1/14.7	*1.37 (1.01–1.86)*	*0.04*	1.01 (0.73–1.40)	0.96
Study duration at home (**<**1 h/**≥** 1 h)	21.5/12.5	*1.89 (1.43–2.50)*	*< 0.001*	*1.82 (1.34–2.49)*	*< 0.001*
Paternal smoke (yes/no)	22.2/14.7	*1.66 (1.29–2.14)*	*< 0.001*	*1.47 (1.12–1.92)*	*< 0.01*
Maternal smoke (yes/no)	31.3/16.3	*2.34 (1.66–3.29)*	*< 0.001*	*1.87 (1.29–2.72)*	*< 0.001*
Subjective SES					
High	13.9	1		*1*	
Middle	18.8	*1.43 (1.04–1.97)*	*0.03*	1.37 (0.99–1.90)	0.06
Low	20.8	*1.62 (1.14–2.32)*	*< 0.01*	*1.48 (1.03–2.14)*	*0.04*

*SES* socioeconomic status, *OR* odds ratio, *CI* confidence interval

^a^All variables were simultaneously included in the multivariate model. The numbers in Italics showed a statistical significant

Table 3 shows the results of logistic regression analyses on low AP stratified by grade. In children in junior grades (1 to 3), fundamental lifestyles such as late wakeup time ≥ 7:00 (OR 3.47; 95% CI, 1.41–8.53), rare physical activity (OR 1.84; 95% CI, 1.09–3.21), studying at home < 1 h (OR 1.81; 95% CI, 1.08–3.02), and both parental smoke (OR 1.67; 95% CI, 1.13–2.48 for father and OR 2.28; 95% CI, 1.32–3.94 for mother) were significantly associated with low AP, while SES was not. In contrast, SES was significantly or marginally associated with low AP in children in senior grades (OR 1.62; 95% CI, 1.02–2.58 for middle SES and OR 1.52; 95% CI, 0.92–2.57 for low SES). Short study duration showed higher OR with low AP than did SES.

**Table 3:** Logistic regression analyses on low academic performance by grade *n* = 1663

	Junior grade (1 to 3)			Senior grade (4 to 6)		
	Low academic	Univariate	Multivariate^a^		Univariate	Multivariate^a^
	%	OR (95% CI)	OR (95% CI)	%	OR (95% CI)	OR (95% CI)
Sex (girl/boy)	15.8/19.3	0.78 (0.55–1.12)	0.79 (0.54–1.16)	19.0/17.8	1.08 (0.76–1.53)	1.30 (0.89–1.89)
Wakeup time on week day						
< 6:30 a.m.	14.7	1	1	14.7	1	1
6:30 to < 7:00	19.5	1.40 (0.97–2.03)	1.27 (0.86–1.89)	21.3	*1.56 (1.08–2.25)*	1.38 (0.94–2.03)
**≥** 7:00	40.0	*3.88 (1.67–9.00)*	*3.47 (1.41–8.53)*	27.5	*2.19 (1.04–4.63)*	1.84 (0.85–4.43)
Skipping breakfast (yes/no)	32.4/16.9	*2.37 (1.16–4.83)*	1.27 (0.56–2.87)	23.3/17.9	1.39 (0.78–2.47)	0.99 (0.53–1.84)
Screen time on week day (≥ 2 h/< 2 h)	23.5/14.4	*1.82 (1.27–2.63)*	1.45 (0.98–2.15)	21.3/16.2	1.40 (0.98–1.98)	1.29 (0.89–1.88)
Physical activity						
Very often	14.1	1	1	17.1	1	
Often	16.6	1.22 (0.77–1.91)	1.49 (0.92–2.40)	20.5	1.25 (0.81–1.92)	1.22 (0.78–1.90)
Rarely	23.8	*1.90 (1.16–3.13)*	*1.84 (1.09–3.12)*	17.0	0.99 (0.62–1.58)	0.99 (0.61–1.62)
Bedtime on week day (late/not late) late: **≥** 10 for junior and **≥** 10:30 for senior grades children	22.3/16.9	1.41 (0.84–2.38)	0.92 (0.51–1.64)	19.7/18.2	1.10 (0.69–1.78)	0.86 (0.51–1.46)
Daytime sleepiness (yes/no)	23.6/16.7	1.54 (0.95–2.52)	1.41 (0.83–2.40)	19.4/18.1	1.09 (0.71–1.66)	0.98 (0.63–1.53)
Obesity (yes/no)	20.6/17.3	1.24 (0.67–2.30)	1.00 (0.52–1.93)	20.0/18.3	1.12 (0.59–2.10)	1.17 (0.60–2.27)
Cram school (no/yes)	18.6/14.2	1.38 (0.87–2.17)	1.01 (0.62–1.66)	19.6/15.0	1.38 (0.91–2.09)	1.03 (0.66–1.61)
Study duration at home (< 1/**≥** 1 h)	19.7/11.1	*1.98 (1.23–3.19)*	*1.81 (1.08–3.02)*	23.5/13.2	*2.02 (1.41–2.90)*	*1.92 (1.29–2.85)*
Paternal smoke (yes/no)	23.3/13.4	*1.96 (1.37–2.82)*	*1.67 (1.13–2.48)*	21.2/16.0	1.41 (0.99–2.00)	1.27 (0.87–1.84)
Maternal smoke (yes/no)	35.7/15.5	*3.03 (1.86–4.94)*	*2.28 (1.32–3.94)*	27.6/17.2	*1.83 (1.13–2.97)*	1.56 (0.92–2.64)
Subjective SES						
High	14.5	1	1	13.3	*1*	*1*
Middle	17.5	1.25 (0.80–1.95)	1.15 (0.72–1.84)	20.2	*1.65 (1.04–2.59)*	*1.62 (1.02–2.58)*
Low	21.3	1.59 (0.97–2.62)	1.38 (0.82–2.34)	20.4	*1.66 (1.00–2.77)*	1.52 (0.92–2.57)

*SES* socioeconomic status, *OR* odds ratio, *CI* confidence interval

^a^All variables were simultaneously included in the multivariate model. The numbers in Italics showed a statistical significant

As an additional test, we demonstrated lifestyle differences by SES (Table 4). Children of lower SES tended to watch screen longer, stay up late, and feel sleepy and were less likely to go to cram school. There was no significant trend in parental smoke in terms of family affluence.

**Table 4:** Subjective SES and lifestyles *n* = 1663

	Subjective SES (%)			Chi-square	Trend
	High	Middle	Low	*p*	*p*
Wakeup time on week day **≥** 7:00 a.m.	3.9	3.6	4.4	0.55	0.59
Skipping breakfast (yes)	5.0	6.8	8.1	0.18	0.07
Screen time on week day (≥ 2 h)	*34.2*	*40.1*	*41.9*	0.04*	0.02*
Physical activity					
Very often	28.1	28.0	29.9	0.82	0.86
Often	46.0	43.6	43.4		
Rarely	25.9	28.4	26.7		
Late bedtime on week day (**≥** 10 for junior, and **≥** 10:30 for senior grades children)	*10.9*	*13.4*	*15.7*	*0.11*	0.04*
Daytime sleepiness (yes)	*12.6*	*17.3*	*20.8*	< 0.01*	< 0.01*
Obesity (yes)	7.2	8.4	8.1	0.74	0.61
Cram school (yes)	*29.0*	*24.2*	*22.1*	*0.05*	0.02*
Study duration at home (**<** 1 h)	61.0	61.1	66.9	0.10	0.08
Paternal smoke (yes)	42.0	45.6	43.9	0.47	0.56
Maternal smoke (yes)	8.5	11.7	12.3	0.14	0.07

Chi-square and trend test

*SES* socioeconomic status

*p < 0.05. The numbers in Italics showed a statistical significant

## Discussion

We demonstrated that 18% of children reported having subjectively low AP, which was associated with late wakeup time, prolonged screen time, short study duration at home, both parental smoke, and low SES. Moreover, our findings showed that though SES was significantly associated with AP among elementary school children, wakeup and study habits had stronger associations with low AP than did SES. In stratified analyses, fundamental lifestyles including sleep, physical activity, and study habits, and both parental smoke were significantly associated with low AP in younger children, while in older children, only study duration and lower SES were associated with low AP. Stronger associations between AP and SES were seen in senior (grades 4 to 6) than in junior children.

### Basic characteristics

Compared to the national survey showing that about 30% of elementary school children had subjectively poor AP [29], the proportion in our study was a little lower (18%). In fact, the average scores of children in this prefecture in the national exam have been slightly higher than the national average [30]. Regional academic atmosphere and teachers’ attitudes might affect children’s AP. In our study, the prevalence of low AP did not differ greatly by sex. Some previous studies showed that girls were likely to have better AP than boys [8, 31, 32], but results are inconsistent. Faught et al. reported that girls had better scores in writing and reading but not in mathematics [6]. The fact that our questionnaire considered subjective AP in general and did not take into account objective scores for each subject could explain why sex differences in AP were not generated.

### Breakfast and sleep

Many previous studies have shown that dietary habits such as skipping breakfast and vegetable intake are associated with low AP [7, 9]. Although there was a significant association in the univariate analysis, the association disappeared in the multivariate analysis. The possible explanations for this are that only a small proportion of children in our study skipped breakfast and that there might be a correlation between skipping breakfast and wakeup time, that is, children who wake up later tend to skip breakfast.

The association between sleep and AP among children has previously been demonstrated [33, 34]. A review by Dewald et al. showed that sleepiness and insufficient sleep were associated with low AP [34]. However, in our study, bedtime and daytime sleepiness did not have a significant association with low AP. A possible reason for this difference is that there might be a curvilinear trend: children who stay up late and experience daytime sleepiness are likely to study till late at night. Actually, in a study from 1125 Japanese 4th-grade children, Oka et al. reported that those with both low and high AP tended to go to bed later than those with average AP [35]. This might nullify the association between bedtime, daytime sleepiness, and AP. In contrast, late wakeup was strongly associated with low AP in this study. In the past, there have been studies to assess the relationship of morning preference with children’s AP [35, 36]. Gaina et al. reported that children with evening preference tended to have longer sleep latency and felt worse in the morning than those with morning preference [37]. It is probable that morning preference affects motivation and subjective AP. Thus, we suggest the importance of morning preference as a contributing factor to school performance among elementary school children.

### Screen time and physical activity

There have been several studies describing the association between screen time, physical activity, and AP [6, 9, 31]. Similarly, two or more hours of screen time was significantly associated with low AP in our study, even after adjusting for other aspects of lifestyle and family environments. Our findings can support the proposal from the Japan Pediatric Association to limit screen time to 2 h a day to lead a healthy life [21], as well as to prevent low AP for elementary school children.

With regard to physical inactivity, the results of the association with AP in previous studies have been inconsistent. Although several studies showed a significant association [28, 31, 38], Faught et al. reported no association between physical activity and AP in grade 5 students [6]. In our study, while there was no significant association overall, the grade-stratified analyses demonstrated a significant association among children in junior grades and no association in senior grades. A plausible explanation for the lack of association in children in senior grades may be that SES has more of an influence on AP in senior grades than it does in junior grades, via going to quality cram school or purchasing additional textbooks. As a result, physical activity might not correlate with children’s AP in senior grades.

### Parental smoke

Prenatal and postnatal secondhand smoke exposure were shown to be associated with poor cognitive parameters in children [17, 39]. A significant inverse relationship between serum cotinine level and reading and math scores has been reported [40]. In line with such studies, our results showed that both parental smoke was significantly associated with low AP, especially in the context of maternal smoke. Although the mechanisms by which secondhand smoke exerts its effects on cognitive functions are still unknown, two substances—carbon monoxide that may deplete oxygen supply to the brain and nicotine or cotinine that would affect both the survival and growth of essential nervous system components—are thought to cause low mental performance [41, 42]. Our findings pose important public health implications. In Japan, governmental tobacco control measures target mainly public places such as schools, hospitals, restaurants, and public transport, not individual homes [18]. To reduce children’s secondhand smoke exposure, additional policy measures are needed.

Analyses stratified by grade showed that in children in junior grades (1 to 3), parental smoke was strongly associated with low AP. This might stem from the amount of time spent with parents at that age. Padrón et al. demonstrated a dose-response relationship between secondhand smoke exposure and psychological distress in adolescents; those exposed to secondhand smoke for less than 1 h at a time did not display an association with psychological distress [15]. Our findings may be explained by the fact that children in junior grades spend more time with their parents, especially their mothers, than do those in senior grades. Health care providers, parents, and policymakers should be aware of younger children’s vulnerability to secondhand smoke.

### Socioeconomic status

We demonstrated that SES was inversely associated with low AP, even after adjusting for sex, grade, lifestyles, and parental smoke. Our findings were in accordance with previous studies from other countries [10, 16, 43]. To date, there have been few studies clarifying children’s AP and SES in Japan [13, 14], and the ones that have been conducted have focused on learning-related activity such as attending cram school, visiting museums, and parental expectations. To the best of our knowledge, this is the first study to comprehensively clarify the association between children’s lifestyles, parental smoke, SES, and AP in Japan.

From the grade-stratified analyses, it emerged that the association between low AP and SES was stronger in senior than in junior grades. This can support the Nippon Foundation report demonstrating that the academic difference due to SES widened in children aged 10 or older [14]. In addition, we showed that late wakeup and short study duration at home had higher ORs with low AP than did SES (Table 2). These findings can encourage children belonging to less affluent households. From the additional analysis, children of lower SES were more likely to have long screen time, stay up late, and feel sleepy in the daytime (Table 4). These unhealthy lifestyles may partly account for the academic inequality. Teachers and health providers should help all children establish regular, morning preference lifestyles and limit screen time. Parents need to understand the harm secondhand smoke causes to children.

### Strengths and limitations

This study involved elementary school children in a relatively homogeneous setting, owing to the lack of private schools in the prefecture. All children attend public school, thereby that can enhance the generalizability of our findings. The high response rate by children, as well as parents, should be considered as strength. We conducted a rare survey to comprehensively assess the association of low AP among school children with their lifestyles, parental smoke, and SES in Japan. However, our study has some limitations. First, SES was evaluated by only one questionnaire: subjective family affluence. This is because it is still hard to obtain SES variables, such as annual household income and parental educational level and occupation, in the school setting in Japan [12]. Instead, subjective affluence has been used in many Japanese studies [44, 45]. Further studies should use objective data to assess the economic status and children’s AP in Japan. Second, our study design was cross-sectional, so we were not able to infer causation. Longitudinal studies will be needed to clarify the effect of parental smoke, SES, and children’s lifestyles on their AP. Third, we cannot discount the possibility of a selection bias with regard to the children included in and excluded from our analysis (1663, 78.1% and 466, 21.9%, respectively), which might attenuate the association of subjective AP and independent variables because the prevalence of low AP, low SES, screen time ≥ 2 h, and maternal smoke were higher in excluded children. Our findings should be treated with caution as the associations of AP with these variables might have been underestimated.

## Conclusions

Our study provides data about low AP and its correlates among Japanese elementary school children. Short study duration at home, late wakeup, prolonged screen time, parental smoke, and low SES are independently associated with low AP. Short study duration and late wakeup were more strongly associated with low AP than was low SES. Moreover, both parental smoke also had a significant association. Parents, health practitioners, and policymakers should pay more attention to SES and parental and children’s lifestyle factors to prevent low AP and to close academic inequality.

## Abbreviations

AP: Academic performance
SES: Socioeconomic status
